# The Real-World Usability, Feasibility, and Performance Distributions of Deploying a Digital Toolbox of Computerized Assessments to Remotely Evaluate Brain Health: Development and Usability Study

**DOI:** 10.2196/53623

**Published:** 2024-05-13

**Authors:** Mouna Attarha, Henry Mahncke, Michael Merzenich

**Affiliations:** 1 Posit Science San Francisco, CA United States

**Keywords:** web-based cognitive assessment, remote data collection, neurocognition, cognitive profiles, normative assessment data, brain health, cognitive status, assessment accessibility

## Abstract

**Background:**

An ongoing global challenge is managing brain health and understanding how performance changes across the lifespan.

**Objective:**

We developed and deployed a set of self-administrable, computerized assessments designed to measure key indexes of brain health across the visual and auditory sensory modalities. In this pilot study, we evaluated the usability, feasibility, and performance distributions of the assessments in a home-based, real-world setting without supervision.

**Methods:**

Potential participants were untrained users who self-registered on an existing brain training app called BrainHQ. Participants were contacted via a recruitment email and registered remotely to complete a demographics questionnaire and 29 unique assessments on their personal devices. We examined participant engagement, descriptive and psychometric properties of the assessments, associations between performance and self-reported demographic variables, cognitive profiles, and factor loadings.

**Results:**

Of the 365,782 potential participants contacted via a recruitment email, 414 (0.11%) registered, of whom 367 (88.6%) completed at least one assessment and 104 (25.1%) completed all 29 assessments. Registered participants were, on average, aged 63.6 (SD 14.8; range 13-107) years, mostly female (265/414, 64%), educated (329/414, 79.5% with a degree), and White (349/414, 84.3% White and 48/414, 11.6% people of color). A total of 72% (21/29) of the assessments showed no ceiling or floor effects or had easily modifiable score bounds to eliminate these effects. When correlating performance with self-reported demographic variables, 72% (21/29) of the assessments were sensitive to age, 72% (21/29) of the assessments were insensitive to gender, 93% (27/29) of the assessments were insensitive to race and ethnicity, and 93% (27/29) of the assessments were insensitive to education-based differences. Assessments were brief, with a mean duration of 3 (SD 1.0) minutes per task. The pattern of performance across the assessments revealed distinctive cognitive profiles and loaded onto 4 independent factors.

**Conclusions:**

The assessments were both usable and feasible and warrant a full normative study. A digital toolbox of scalable and self-administrable assessments that can evaluate brain health at a glance (and longitudinally) may lead to novel future applications across clinical trials, diagnostics, and performance optimization.

## Introduction

### Background

There is strong public interest in brain health evaluation, with 9 of 10 citizens expressing an interest in understanding how their brain is functioning [1]. Best practices for remote cognitive and behavioral assessment established by the Alzheimer Society of Canada Task Force [2] and jointly by the American Academy of Clinical Neuropsychology and the National Academy of Neuropsychology [3] recommend that assessments be normed and validated; usable; agnostic to demographic variables such as level of education, race and ethnicity, and gender; and supported by a secure and reliable technical platform.

The field of cognitive evaluation currently offers 3 general classes of cognitive assessment: traditional neuropsychological instruments [4], supervised computerized assessments [5], and unsupervised self-administrable assessments [6].

The traditional approach of assessing cognition [7] is standardized, psychometrically sound, valid and reliable, and diagnostic [8] yet also cumbersome, time limited, and costly. Standard assessment batteries generally require face-to-face administration and manual scoring, limiting test frequency and access for individuals who cannot take time off from work, travel, or afford attendant costs. Assessment administration usually only occurs in the context of a serious cognitive concern, often without a baseline for comparison, and is generally modeled by a one-size fits all approach that infers cognitive decline from deviations from population averages rather than from the decline trajectories capturing individual differences. This frustrates identification of what might otherwise have been clear prodromal indications. Traditional assessments are also generally administered at discrete, infrequent time points that inadequately support interventional clinical trials; inadequately paint a comprehensive picture of fine-grained changes in ability or track the inflections in cognitive trajectories; inadequately document quality-of-life impacts over time; and inadequately serve those with geographical, economic, or physical limitations. For these and other reasons, the traditional assessment administration approach, although valuable, is not optimal for proactive, continuous, early detection or for general brain health monitoring [9].

Several of these challenges are overcome by supervised computerized assessments [10] (eg, the National Institutes of Health [NIH] Toolbox [11], NIH Executive Abilities: Measures and Instruments for Neurobehavioral Evaluation and Research [EXAMINER] [12], Cogstate [13], and CNS Vital Signs [14]). These batteries have strong normative data sets supporting their interpretation, brief testing durations, and automated scoring yet still require administrator training and oversight, device restrictions, or software installation that in some cases prevent widespread scalability, especially in vulnerable populations without access to researcher or clinician support. Furthermore, similar to the traditional approach, the theoretical foundation underlying the design of many of these assessments is often neuropsychological and nonadaptive, emphasizing domains such as list learning and recall, language, fluency, visual-constructional skills, planning, orientation, or episodic memory rather than being neurologically designed to assay the elemental status of brain health.

Several self-administered computerized neurocognitive testing batteries have been developed and validated in recent years, such as the Cambridge Neuropsychological Test Automated Battery [15] and BrainCheck [16], with notable work demonstrating real-world usability and feasibility in naturalistic in-home settings [17,18], validation across technology platforms [19], and validity in specific clinical populations [20,21]. Computerized self-administered cognitive assessments were recently reviewed in a meta-analysis [22]. A total of 10 tools was evaluated, with the authors noting substantial gaps in the size of validation populations, lack of diversity in such populations [22], and few studies supporting the usability and feasibility of such assessments in real-world environments [23].

### Assessment Design Principles

To align assessment development with best practices from the Alzheimer Society of Canada Task Force [2], the American Academy of Clinical Neuropsychology, and the National Academy of Neuropsychology [3], a digital toolbox of assessments should be (1) neurologically informed to show greater sensitivity to disruptions in underlying issues of brain health; (2) usable, scalable, and self-administrable by people in an unsupervised environment, representing a significant advance over the current standard that relies on in-person assessment with a trained clinician in a supervised environment; (3) adaptive, with progressive adjustments in difficulty to more accurately capture the individual’s performance limits; and (4) supported by a robust, reliable, and secure technical infrastructure. These practices will mitigate the challenges of efficient global assessment and advance the science of measuring cognition in natural settings.

#### Neurologically Informed

Assessments that are neurologically informed target the elemental health status of the brain. The most fundamental, system-wide changes that occur with age are a general slowing of processing speed [24], an increase in spatiotemporal receptive field size [25], and an influx of neuronal noise [26], which affect the precision and reliability of accurate perception and cognition [27]. In the case of vision, for example, representations are hierarchically established within separate pathways of the visual system and then integrated to varying degrees upstream based on complexity [28]. Basic visual features—such as orientation [29] and brightness [30]—are generated by mechanisms in early visual stages via feature selective cells [31]. On the other hand, more complex stimuli—such as size [32] and motion [33]—require the integration of multiple-component feature populations and are generated further along the ventral or dorsal pathways or even after the convergence of these pathways, as the case may be for highly complex stimulus sets [34]. Slowed and inaccurate low-level neuronal information processing produces impaired or *noisy* representations that adversely affect the input to the networks upstream that govern more complex cognitive abilities. Motivated by recognition of the system-wide neurological changes that occur with age [25], all assessments in this battery were designed to strongly rely on processing speed, with many also requiring low-level sensory discrimination. In addition, declines in executive function [35-42] are closely associated with functional status [43-48]; therefore, we also designed assessments to assay higher-order cognitive functions such as working memory [12,49], which is known to change across the lifespan [50,51].

#### Usable, Self-Administrable, and Customizable

An efficient assessment system must offer high usability, allowing for a higher intensity and frequency of assessment sessions than would be possible with existing approaches, especially in underserved communities, by meeting the following criteria. Assessments should (1) be self-administrable; (2) be brief; (3) use standardized instructions that are simple to understand; (4) include a tutorial and set of practice trials before test trials; (5) present results that are easily interpretable; (6) minimize or eliminate culture-specific references to the extent possible (eg, letters or numbers); (7) present task instructions in multiple languages; and (8) allow access across devices, including the web, iOS, and Android [3]. This would accommodate routine self-testing for brain health, analogous to at-home blood pressure monitoring for heart health to reduce patient morbidity and mortality [52]. Furthermore, test batteries should be customizable by a clinician, allowing for the use of test batteries ranging from brief screening batteries to deep investigations of target cognitive or neurological domains to broad assessments across multiple functions, much in the same way that a blood panel can be customized to comprise a standard panel of metabolites or specific tests relevant to potential conditions.

#### Adaptive

Assessments should be designed to progressively adapt in difficulty to maintain an optimal level of challenge on a moment-to-moment basis. This allows individuals to progress through the assessment at their own rate, preventing the assessment from being too easy or too difficult. For example, assessments may use a statistically optimal approach (eg, Bayesian or staircase procedure that adapts to approximately 80% criterion accuracy) that allows the assessment to continuously adjust the adaptive dimension of the task to the unique sensory and cognitive capabilities of the user.

#### Supported by a Robust Technical Infrastructure

Assessments should be built on software infrastructure that (1) provides core user management features (eg, account creation and log-in), engagement features to make performing the assessments compelling to users, and flexibility to perform a sequence of assessments in a single session or across multiple days; (2) accommodates granular role-based access control to administrators or clinicians for user oversight via a secure, Health Insurance Portability and Accountability Act–compliant web-based group portal to remotely supervise (and analyze) use, progress, and performance data using either individual or bulk download functions; (3) offers a comprehensive and secure application programming interface [API] allowing for organizational access to user data through a modern web service architecture on demand to integrate data collected onto partner user databases and performance management systems (eg, Epic); (4) accommodates upgrades and review and release processes for web and mobile platform to support new operating system releases; and (5) assumes that complete physical loss happens on an occasional basis and implements a protocol to quickly and fully recover the data.

### This Study

In this study, we developed and deployed a set of brain health assessments using a software infrastructure that supports usability and scalability. Assessments were united in their requirements for speed, accuracy, and adaptivity [53] and were derived from existing brain training exercises on an on-market platform called BrainHQ (developed by Posit Science). Each assessment used stimuli identical to a specific stimulus configuration of an existing exercise and a modified adaptive tracking algorithm. Deriving the assessments from gamified [54], evidence-based brain training exercises is a strength of our approach. To the extent that the training exercises have demonstrated *neurological benefit* (eg, neural timing [55,56], brain activation [57], and functional connectivity [58-60]), *cognitive benefit* (eg, speed [61-63], attention [56,64,65], and memory [62,65,66]), and *functional benefit* (eg, mood [67-70], quality of life [71], health [72-74], driving safety [75,76], balance [77-79], verbal fluency [80], and everyday performance [81-83]), assessments that replicate the task demands of the training exercises should then be able to *evaluate* the same neurological networks, cognitive constructs, and functional abilities that are engaged and improved by their associated brain training exercise.

The Useful Field of View (UFOV) task, now renamed Double Decision on BrainHQ, highlights the close relationship between the neurological, cognitive, and functional benefits of UFOV training versus the neurological, cognitive, and functional underpinnings that the UFOV assessment evaluates. Several hours of UFOV training strengthens functional connectivity in areas associated with cognitive decline (neurological) [84], improves performance on standard tests of memory and executive function (cognition) [85], and reduces the risk of automobile collisions by 48% (function) [76], whereas assessment performance at a single time point using the same task demands shows sensitivity to the integrity of neural networks involved in cognitive decline (neurological) [86], correlates with baseline performance on standard neuropsychological measures of memory and executive function (cognition) [87], and predicts the future likelihood of at-fault motor vehicle collisions in a real-world setting (function) [88]. Assessments that are back engineered from validated training exercises reflect a novel approach to assessment design that complements the standard approach rooted in neuropsychology.

Small subsets of the 29 BrainHQ assessments have been deployed, normed, and validated in recent years. In a pilot study, the assessments were considered feasible, enjoyable, and acceptable [89]. Another study by the same group showed that assessments could distinguish patients with schizophrenia from healthy controls [90], with assessment performance correlating with global cognition in healthy adults, patients with psychosis, and first-degree biological relatives of patients with psychosis [91]. In a fully remote study highlighting the relationship between heart health and brain health, participants with atrial fibrillation showed lower performance on assessments of working memory and episodic memory, whereas participants with hypertension showed lower performance on episodic memory [92].

The aims of this pilot study were to (1) design a set of neurologically informed, adaptive, and usable assessments that can be self-administered on personal devices at home without supervision; (2) deploy the assessments using a robust technical infrastructure on web, iOS, or Android; and (3) evaluate metrics of usability, feasibility, and assessment performance distributions.

## Methods

### Ethical Considerations

The protocol was submitted to the Western Institutional Review Board and received regulatory exemption. Informed consent was waived, and study personnel made no contact with participants who registered. Participants could discontinue at any time by closing their browser. Participants who completed the full battery of 29 assessments received an annual subscription to BrainHQ’s brain training program, which was automatically applied to their account upon completion. No monetary incentives were offered. Data sets were deidentified, and randomly generated unique IDs linked participant performance data with participant demographic information.

### Participant Recruitment

This study used a convenience sample. Between the months of May 2022 and November 2022, we emailed a subset of 365,782 English-speaking commercial users from the BrainHQ database who had originally registered between January 2018 and April 2022 and who had completed an insignificant number of levels on BrainHQ’s training program (0-10 levels). All users were nonpaying members of the database.

### Assessment Selection and Design

The battery comprised 29 assessments designed to evaluate core indexes of brain health across the visual and auditory sensory modalities (Textbox 1). Each assessment was derived from one of the levels from each of the 29 brain training exercises offered through BrainHQ. The level that was selected had a reasonable performance histogram (via visual inspection) from trained commercial users and used stimulus parameters (such as contrast, eccentricity, discriminability, and speech speed) that were of medium difficulty relative to other levels presented within the exercise. The underlying algorithm for the assessment was then modified to base the exit criteria on asymptotic performance (rather than exit based on the preset trial count used in training). A tutorial video of the general task requirements for each assessment is provided on BrainHQ’s YouTube channel [93].

**Commercial names of the 29 brain health assessments that participants in this remote pilot study self-administered at home on a personal, internet-connected device without supervision. Assessment descriptions include the experimental paradigm (where applicable), sensory modality, task requirement, and adaptive dimension.**

**Assessment name and description**

- Double Decision: in a dual-task paradigm assessing Useful Field of View, participants discriminate a visual stimulus presented in the center of gaze while simultaneously locating a target in the peripheral visual field. The adaptive dimension is display exposure duration.
- Target Tracker: in a speeded multiple object–tracking paradigm, participants track a set of targets (defined by their spatiotemporal onset) among visually identical distractors. The adaptive dimension is set size (the number of objects tracked).
- Mixed Signals: in an audiovisual Stroop paradigm, participants listen to auditory information and determine whether flanked visual information presented on-screen is an exact match. The adaptive dimension is display exposure duration.
- Freeze Frame: in a reverse go/no-go paradigm, participants remember a target image presented at the start of the trial, after which a continuous stream of targets and foils are interleaved with unequal probability. Users withhold a motor response to all targets. The adaptive dimension is target and foil frequency.
- Divided Attention: in a continuous performance paradigm, users rapidly determine whether flashing colors, shapes, or patterns meet a prespecified rule. The adaptive dimension is display exposure duration.
- Hear, Hear 2: in a memory-based auditory distractor suppression paradigm, participants remember a single target tone at the start of the assessment and report whether sets of foil tones of increasing similarity contain the target. The adaptive dimension is similarity of the foil tones to the target tone.
- Mind’s Eye: in a visual distractor suppression paradigm, participants remember the orientation of a set of moving dots and report whether a set of similar images presented contains the target. The adaptive dimension is similarity of the foil orientations to the target orientation.
- Memory Grid: participants visuospatially match identical cards representing confusable syllables. The adaptive dimension is set size (the number of syllable pairs to match).
- Rhythm Recall: participants listen to tonal beats played over a melody and later replay how long each beat was played and where in the melody the beats changed. The adaptive dimension is set size (the number of beats remembered).
- Scene Crasher: in a change detection paradigm, participants select the item added to a visual scene. The adaptive dimension is set size (the number of nontargets in the visual scene).
- Syllable Stacks: in a span paradigm, participants report the order of presented confusable syllables in a serial memory span task. The adaptive dimension is set size (the number of syllables remembered).
- To-Do List Training: participants hear a sequence of instructions that must be retained over a delay and recall those items by selecting items in order from a visual grid that includes targets, distractors, and foils. The adaptive dimension is set size (the number of instructions remembered in sequence).
- Eye For Detail: participants identify the locations of identical targets among a variable number of distractors. The adaptive dimension is display exposure duration.
- Fine Tuning: participants indicate which of 2 confusable syllables was presented. The adaptive dimension is stimulus similarity.
- Hawk Eye: in a visual search paradigm, participants identify the location of a target among distractors. The adaptive dimension is display exposure duration.
- Sound Sweeps: in a time-order-judgment paradigm, 2 successive frequency-modulated tone sweeps are presented, and participants indicate whether the frequency increased or decreased within each tone. The adaptive dimension is sweep speed.
- Visual Sweeps: in a time-order-judgment paradigm, 2 drifting gratings are presented, and participants indicate the direction of drift for each grating. The adaptive dimension is drift speed.
- Face Facts: participants remember a set of facts associated with a person. The adaptive dimension is set size (the number of person-factoid associations remembered).
- Face To Face: participants select the face with the same emotional expression as a target face presented previously. The adaptive dimension is display exposure duration.
- In The Know: participants listen to a conversation and recall facts through a series of multiple-choice questions. The adaptive dimension is set size (the number of subtopics discussed).
- Recognition: participants select the face with the identity of a target face presented previously. The adaptive dimension is display exposure duration.
- Mental Map: in an egocentric spatial mental rotation task, participants remember the relative location of objects in a grid and then reconstruct the grid from memory after it has been rotated, flipped, or translated. The adaptive dimension is complexity of the scene transformations.
- Optic Flow: in a visuomotor paradigm, participants view a road scene and make rapid visual discriminations in the center of gaze while staying alert to potential hazards in the periphery. The adaptive dimension is display exposure duration.
- Right Turn: in a mental rotation paradigm, participants report whether images in a set are identical or mirror images. The adaptive dimension is display exposure duration.
- True North: in an allocentric spatial mental rotation task, participants remember directions while the cardinal orientation of the scene is manipulated. The adaptive dimension is set size (the number of directions remembered).
- Auditory Ace: in an auditory n-back paradigm, participants report whether the current stimulus matches the stimulus presented *n* steps earlier in the sequence. The adaptive dimension is set size (the number of cards recalled *n* step back).
- Card Shark: in a visual n-back paradigm, participants report whether the current stimulus matches the stimulus presented *n* steps earlier in the sequence. The adaptive dimension is set size (the number of *n* back).
- Juggle Factor: in a visual span paradigm, participants report the order of highlighted discs as they spatiotemporally move in concentric rings. The adaptive dimension is set size (the number of discs remembered).
- Mind Bender: in a task-switching paradigm, participants make decisions on competing stimuli based on changing rules. The adaptive dimension is display exposure duration.

### Flow

**Figure 1:**
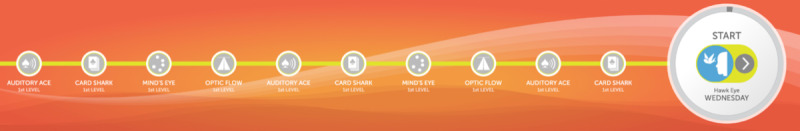
Example of the assessment queue for a registered participant. The program automatically guided participants through each of the 29 assessments, starting with written instructions; a set of practice trials; and, finally, participant-initiated launch of the assessment.

Participants registered by clicking on an embedded link within a recruitment email (Figure S1 in Multimedia Appendix 1) and logged into the website using their established log-in credentials (username and password). Upon log-in, the participants completed a set of demographic questions on age, gender, race and ethnicity, and highest level of education attained (Figure S2 in Multimedia Appendix 1). Participants were randomized to 1 of 4 counterbalanced assessment sequences and then guided through each of the 29 BrainHQ assessments (Figure 1). Each assessment began with a tutorial that included a brief written description of the task followed by several practice trials, after which participants could begin the assessment or replay the tutorial. An assessment ended when the participant (1) reached asymptotic or near-asymptotic performance using an adaptive algorithm, (2) reached the maximum possible score 3 times consecutively (ceiling performance), or (3) reached the worst possible score 3 times consecutively (floor performance). Participants were given their score (a raw score and percentile) after completing each assessment (Figure S3 in Multimedia Appendix 1). They had 3 weeks (21 days) to complete the full battery and could complete the assessments at their own pace within this time frame. Engagement emails were sent automatically on a weekly basis to registered users on days 1, 7, 14, and 20 (Figure S4 in Multimedia Appendix 1).

### Demographics Questionnaire

Participants who registered for the study saw a web-based questionnaire of 4 questions that appeared automatically upon a password-protected log-in to their existing accounts (Figure S2 in Multimedia Appendix 1). The prompt for age was “How old are you? I was born in:” with a number scroll presenting years from 1907 to 2012. The prompt for gender was “What is your gender? I identify as:” with response options for “female,” “male,” “non-binary,” or “other.” The prompt for education was “What is the highest level of education you have received?” with response options suggested by the NIH Common Data Elements Repository: “never attended,” “kindergarten,” “elementary,” “middle school,” “high school or GED,” “some college/no degree,” “associate degree or vocational program,” “bachelor’s degree,” “master’s degree,” or “doctoral or professional degree.” The prompt for race and ethnicity was “What is your ethnicity? Enter all that apply” with response options suggested by the NIH Common Data Elements Repository: “American Indian or Alaska Native,” “Asian,” “Black or African American,” “Hispanic or Latino,” “Native Hawaiian or Other Pacific Islander,” or “White.” Questions could be declined by withholding a response and pressing “continue.” The usability and technical functionality of the questionnaire were tested by a quality assurance team before deployment.

Questions were presented one at a time (one per screen) in the aforementioned fixed order. No automated completeness checks were conducted as advancement through the questionnaire required (1) a selection of one of the response options or (2) a decline to respond by clicking “continue.” Respondents could change their answers to previous questions (by clicking on a back arrow) within the period between launching the first question and clicking “submit” on the fourth question. No additional review of responses was provided before submission. Unique visitors were determined based on the user’s unique ID, and the survey was presented only once.

### Technical Infrastructure

The BrainHQ assessments leverage Health Insurance Portability and Accountability Act–compliant and System And Organization Controls Type 2 security–certified software infrastructure that was developed for the commercial BrainHQ brain training program and tailored to assessment delivery, management, and recording. The program comprises a wrapper (which manages user log-in, assessment schedules, and user engagement features) and a set of assessments. The program is hosted by Amazon Web Services and is robust to interruptions in connectivity, supporting participants who reside in remote regions.

### Data Analysis

#### Overview

We pulled the assessment data from the BrainHQ server on February 21, 2023. For all analyses, a *P* value of <.05 determined statistical significance. No corrections were made for multiple comparisons, and no outliers were excluded for these exploratory analyses.

#### Demographic Characteristics

All registered participants completed the 4-item demographics questionnaire (age, gender, educational level, and race and ethnicity). We tallied the number of participants who selected a response option to each question and divided it by the total number of participants who registered (N=414). We included both raw numbers and percentages. The arithmetic mean, SD, and range were given for continuous variables (age). Statistical corrections or weights were not used.

#### Completion Rates

The raw number of participants and the percentage (raw out of the total number of registered participants) were calculated for participants who completed 1 assessment and all assessments. Additional completion rates (raw number and percentage) were provided in bins across the 29 total assessments, with the bins defined as 1 to 5 assessments, 6 to 10 assessments, 11 to 15 assessments, 16 to 20 assessments, 21 to 25 assessments, and 26 to 29 assessments.

#### Assessment Characteristics

To evaluate usability, we reported general descriptive statistics (measures of central tendency, such as the arithmetic mean, SD, median, and mode), distribution characteristics (skew and kurtosis), and psychometric properties (performance histograms of the proportion of participants as a function of assessment score, the mean number of minutes spent in each assessment, and the percentage of participants obtaining the numerically lowest or highest assessment score to indicate the frequency of ceiling and floor effects).

#### Associations Between Performance and Age, Gender, Years of Education, and Race and Ethnicity

To establish the association between performance and demographic variables, we used the Spearman ρ for age, Wilcoxon rank sum test for gender (male vs female), Wilcoxon rank sum test for race (White vs people of color), and Spearman ρ for educational level after participant responses were transformed from highest level of education attained to years of education.

#### Composite z Score for “Completers”

Assessment “completers” were defined as participants who finished the full battery of 29 assessments. For this subset of participants, we transformed their raw score on each assessment to a *z* score. We created a composite score by averaging all *z* scores from the 29 individual assessments for each participant. We present the mean *z* score, SD, and range.

#### Cognitive Profiles

Using the *z* scores for assessment completers, we defined an a priori set of 4 basic cognitive profiles informed in part by observations using the brain training analogs of the assessments [94]: a high performer who performed above the mean on all or most tasks, a low performer who performed below the mean on all or most tasks, a strong auditory performer who performed above the mean on auditory assessments but below the mean on visual assessments, and a strong visual performer who performed above the mean on visual assessments but below the mean on auditory assessments. High performers were defined as users with a mean *z* score composite of >0.40. Low performers were defined as users with a mean *z* score composite of <–0.40. The *z* scores for the auditory and visual exercises were averaged separately for each user, and those with a difference of half an SD or more between their mean auditory *z* score and mean visual *z* score were considered to have differential skills across these 2 sensory modalities.

#### Principal Component Analysis

The *z* score transformed data were subjected to exploratory principal component analysis using varimax rotation, and factor loadings of ≥0.40 were identified. The Bartlett sphericity test and Kaiser-Meyer-Olkin assumptions were confirmed.

#### Identifying Assessments With Favorable Properties

Assessments with favorable properties that may be combined to establish a core battery were defined as assessments that (1) load onto different factors from the principal component analysis; (2) scale with age, suggesting that they are sensitive to networks involved in age-related cognitive decline; (3) are agnostic to differences in gender, race, and level of education attained; and (4) show good usability without notable ceiling or floor effects either currently or with future score boundary modifications.

## Results

Of the 365,782 potential participants who were contacted via the recruitment email, 414 (0.11%) registered for the study.

### Participant Demographic Characteristics

Registered participants (N=414) had a mean age of 63.6 (SD 14.8; range 13-107) years and were mostly female (n=265, 64%), with approximately half (n=144, 34.8%) as many male participants and several identifying as nonbinary (n=2, 0.5%) or other (n=1, 0.2%). The sample was relatively educated, with approximately 80% attaining a degree, including a bachelor’s degree (132/414, 31.9%), master’s degree (114/414, 27.5%), doctoral or professional degree (42/414, 10.1%), and associate’s or vocational degree (41/414, 9.9%), and approximately 20% of the sample reporting some college education (45/414, 10.9%), high school education or General Educational Development (26/414, 6.3%), middle school education (3/414, 0.7%), elementary school education (3/414, 0.7%), and having never attended school (8/414, 1.9%). The sample was predominately White (349/414, 84.3%), with approximately 12% representing minority groups such as American Indian or Alaska Native (1/414, 0.2%), Asian (20/414, 4.8%), Black or African American (10/414, 2.4%), Hispanic or Latino (13/414, 3.1%), and ≥2 people of color (4/414, 1%) and 4.1% (17/414) of the participants declining to respond. Table 1 presents the distribution of responses for these demographic variables.

**Table 1:** Responses to demographic questionnaire for registered participants (N=414).

Characteristic		Values
Age (y), mean (SD)		63.6 (14.8)
**Age (y), n (%)**		
	<20	5 (1.2)
	20-29	5 (1.2)
	30-39	18 (4.3)
	40-49	46 (11.1)
	50-59	74 (17.9)
	60-69	121 (29.2)
	70-79	105 (25.4)
	80-89	34 (8.2)
	90-99	5 (1.2)
	100-109	1 (0.2)
**Gender, n (%)**		
	Female	265 (64)
	Male	144 (34.8)
	Nonbinary	2 (0.5)
	Other	1 (0.2)
	Declined to respond	2 (0.5)
**Educational level, n (%)**		
	Bachelor’s degree	132 (31.9)
	Master’s degree	114 (27.5)
	Some college, no degree	45 (10.9)
	Doctoral or professional degree	42 (10.1)
	Associate’s degree or vocational program	41 (9.9)
	High school or GED^a^	26 (6.3)
	Never attended school	8 (1.9)
	Middle school	3 (0.7)
	Elementary school	3 (0.7)
**Race and ethnicity, n (%)**		
	American Indian or Alaska Native	1 (0.2)
	Asian	20 (4.8)
	Black or African American	10 (2.4)
	Hispanic or Latino	13 (3.1)
	Native Hawaiian or other Pacific Islander	0 (0)
	White	349 (84.3)
	≥2 races excluding White	4 (1)
	Declined to respond	17 (4.1)

^a^GED: General Educational Development.

### Completion Rates

Of the 414 participants who registered, 367 (88.6%) completed at least one assessment and 104 (25.1%) completed all 29 assessments. Of those 367 participants, 171 (46.6%) completed 1 to 5 assessments, 41 (11.2%) completed 6 to 10 assessments, 24 (6.5%) completed 11 to 15 assessments, 18 (4.9%) completed 16 to 20 assessments, 5 (1.4%) completed 21 to 25 assessments, and 108 (29.4%) completed 26 to 29 assessments.

### Assessment Characteristics

**Figure 2:**
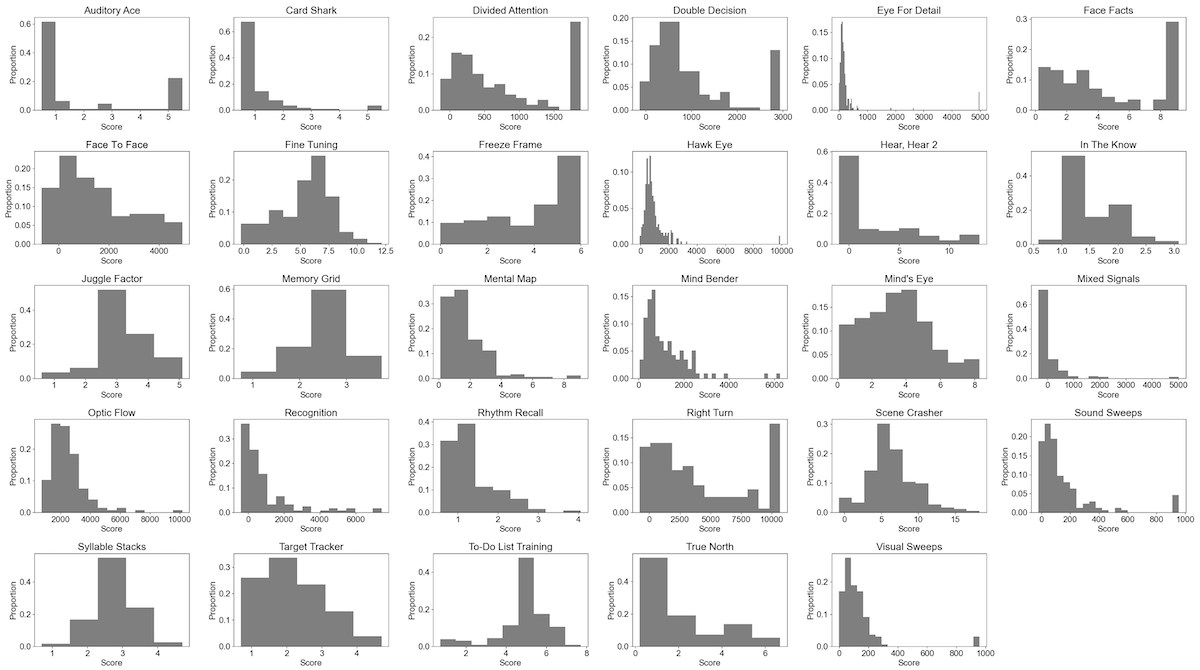
Performance histograms of the 29 assessments presenting the proportion of registered participants who achieved each score defined by the assessment’s adaptive dimension.

Table 2 presents the number of participants who completed the assessments, arithmetic mean, SD, median, mode, skew, kurtosis, absolute number and percentage of participants with ceiling or floor scores, and number of minutes spent on each assessment. Performance histograms for each assessment are presented in Figure 2. The overall mean duration was 3.4 (SD 1.0) minutes. An analysis of the percentage of participants who obtained the numerically lowest score or the numerically highest score showed that 59% (17/29) of the assessments did not have ceiling or floor effects of ≥10%. Of the 12 assessments with ceiling or floor effects, 2 (17%; Auditory Ace and Hear, Hear 2) are unmodifiable and uninformative and should be deprecated, 6 (50%; Card Shark, Freeze Frame, Mixed Signals, Rhythm Recall, Target Tracker, and True North) are unmodifiable but may have diagnostic value (eg, the ability to remember only 1 item may indicate cognitive impairment), and 4 (33%; Divided Attention, Double Decision, Face Facts, and Right Turn) have score bounds that can easily be modified (in this case increased) to eliminate their floor effects.

**Table 2:** Descriptive statistics, distribution characteristics, and psychometric properties to evaluate the general usability of the 29 self-administered assessments deployed in this remote pilot study.

Assessment name	Total number of participants, N	Values, mean (SD)	Values, median (IQR)	Values, mode	Skew	Kurtosis	Highest score, n (%)	Lowest score, n (%)	Time (min), mean (SD)
Auditory Ace^a^	112	2.4 (2.1)	1 (1-3.8)	1	1.0	–0.8	25 (22.3)	65 (58)	2.9 (2.1)
Card Shark^a^	117	1.5 (1.0)	1 (1-1.6)	1	2.9	9.0	3 (2.6)	72 (61.5)	2.4 (2)
Divided Attention^b^	210	928.5 (718.5)	653 (338.5-1679.5)	2048	0.6	–1.2	51 (24.3)	1 (0.5)	2.3 (2.3)
Double Decision^b^	177	1174.6 (890.9)	869 (574-1315)	3162	1.3	0.5	23 (12.9)	1 (0.6)	2.9 (1.3)
Eye For Detail^b^	228	373.4 (913.2)	162 (112-234)	148	4.7	20.6	8 (3.5)	3 (1.3)	3.6 (1.5)
Face Facts^a^	113	5.5 (3.3)	4.5 (2.5-10)	10	0.3	–1.5	31 (27.4)	1 (0.9)	6.1 (3.8)
Face To Face^b^	188	2243.1 (1515.3)	1862 (1000-3236)	5623	0.7	–0.5	11 (5.9)	1 (0.5)	3.9 (1.6)
Fine Tuning^a^	236	6.6 (2.4)	6.9 (5.2-8.2)	6.8	–0.4	–0.2	1 (0.4)	5 (2.1)	1.4 (2)
Freeze Frame^a^	166	4.6 (2.0)	5 (3-6)	7	–0.4	–1.1	40 (24.1)	16 (9.6)	4.6 (0.8)
Hawk Eye^b^	253	1064.1 (1140.1)	794 (562-1189)	750	5.8	42.0	3 (1.2)	1 (0.4)	2.3 (1.1)
Hear, Hear 2^a^	164	4 (3.9)	1.4 (1-6.8)	1	1.2	0.3	1 (0.6)	77 (47)	3.2 (1.6)
In The Know^a^	112	1.9 (0.5)	1.5 (1.5-2.5)	1.5	0.8	–0.1	2 (1.8)	3 (2.7)	2.2 (1.4)
Juggle Factor^a^	146	4 (0.9)	4 (3.5-4.5)	3.5	–0.3	0.6	2 (1.4)	5 (3.4)	4.5 (2.3)
Memory Grid^a^	133	3.2 (0.6)	3.5 (2.8-3.5)	3.5	–0.7	0.5	3 (2.3)	5 (3.8)	4.5 (2.1)
Mental Map^a^	180	2.6 (1.4)	2.5 (1.5-3.5)	1.5	2.2	7.5	2 (1.1)	11 (6.1)	3.8 (2)
Mind Bender^b^	117	1356.7 (983.5)	1082 (685.5-1783)	622	2.3	7.9	1 (0.9)	1 (0.9)	2.6 (1)
Mind’s Eye^a^	150	4.4 (1.9)	4.4 (3-5.5)	1	0.3	–0.3	2 (1.3)	12 (8)	2.9 (0.7)
Mixed Signals^b^	161	382.1 (729.0)	90 (32-476)	32	4.4	23.9	1 (0.6)	64 (39.8)	3.2 (0.9)
Optic Flow^b^	147	3114.8 (1239.7)	2896 (2521-3694)	2896	2.6	10.7	1 (0.7)	1 (0.7)	2.7 (4.8)
Recognition^b^	122	1326.5 (1566.8)	709.3 (437-1488.8)	617	2.4	5.8	2 (1.6)	1 (0.8)	3.3 (1.4)
Rhythm Recall^a^	133	1.6 (0.7)	1.5 (1-2)	1.5	1.4	1.9	1 (0.8)	40 (30.1)	4.3 (1.3)
Right Turn^b^	129	5027.9 (3923.7)	3984 (1833-8457.5)	11,585	0.6	–1.1	23 (17.8)	2 (1.6)	4.5 (1.6)
Scene Crasher^a^	183	8.2 (3.2)	7.8 (6.2-9.8)	8.2	0.6	1.2	1 (0.6)	6 (3.3)	3.6 (1.1)
Sound Sweeps^b^	175	192.5 (209.9)	126 (73-224)	126	2.7	7.5	8 (4.6)	1 (0.6)	2.1 (0.8)
Syllable Stacks^a^	120	3.6 (0.6)	3.5 (3.5-4)	3.5	0.1	1.6	1 (0.8)	1 (0.8)	2.8 (0.9)
Target Tracker^a^	158	2.9 (1.0)	2.9 (2-3.5)	2.5	0.4	–0.3	5 (3.2)	29 (18.4)	3.9 (1.8)
To-Do List Training^a^	132	5.6 (1.2)	5.5 (5.5-6.5)	5.5	–1.3	2.8	1 (0.8)	3 (2.3)	4.5 (2.7)
True North^a^	110	3.3 (1.8)	2.5 (1.8-4.5)	1.5	0.9	–0.1	3 (2.7)	25 (22.7)	4.3 (3)
Visual Sweeps^b^	164	169.5 (161.5)	130 (90-191.9)	86	4.1	18.4	5 (3.1)	1 (0.6)	2.3 (0.6)

^a^Higher scores reflect better performance.

^b^Lower scores reflect better performance.

### Associations Between Performance and Age, Gender, Years of Education, and Race and Ethnicity

**Figure 3:**
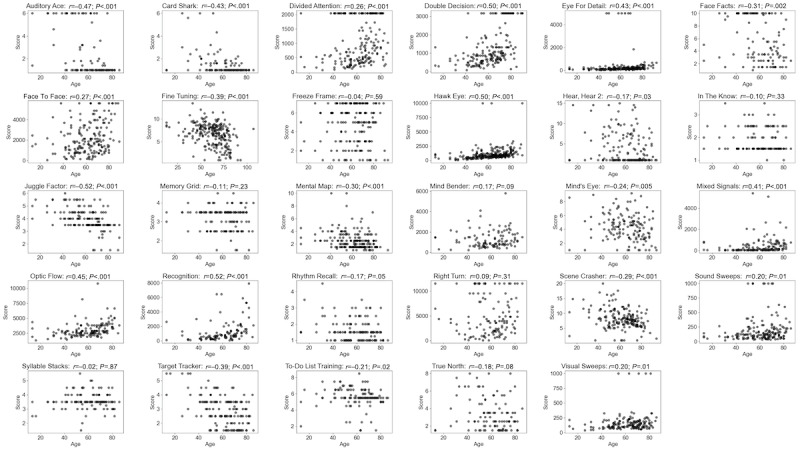
Association between performance and age for each assessment across registered participants.

The relationship between performance and age was statistically significant for 72% (21/29) of the assessments (*P*<.05 in all cases), with the 8 exceptions being Freeze Frame (*P*=.59), In The Know (*P*=.33), Memory Grid (*P*=.23), Mind Bender (*P*=.09), Rhythm Recall (*P*=.05), Right Turn (*P*=.31), Syllable Stacks (*P*=.87), and True North (*P*=.08; Figure 3).

**Figure 4:**
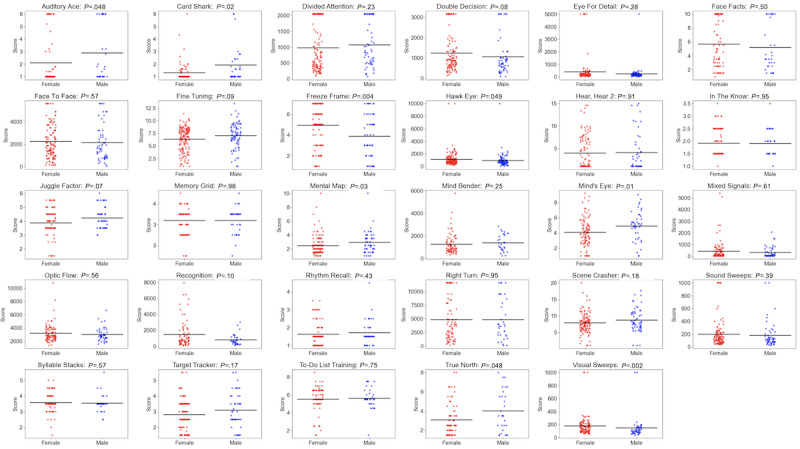
Association between performance and gender for each assessment across registered participants.

The relationship between performance and gender was not statistically significant for 72% (21/29) of the assessments (*P*>.05 in all cases), with the 8 exceptions being Auditory Ace (*P*=.048), Card Shark (*P*=.02), Freeze Frame (*P*=.004), Hawk Eye (*P*=.049), Mental Map (*P*=.03), Mind’s Eye (*P*=.008), True North (*P*=.048), and Visual Sweeps (*P*=.002; Figure 4).

**Figure 5:**
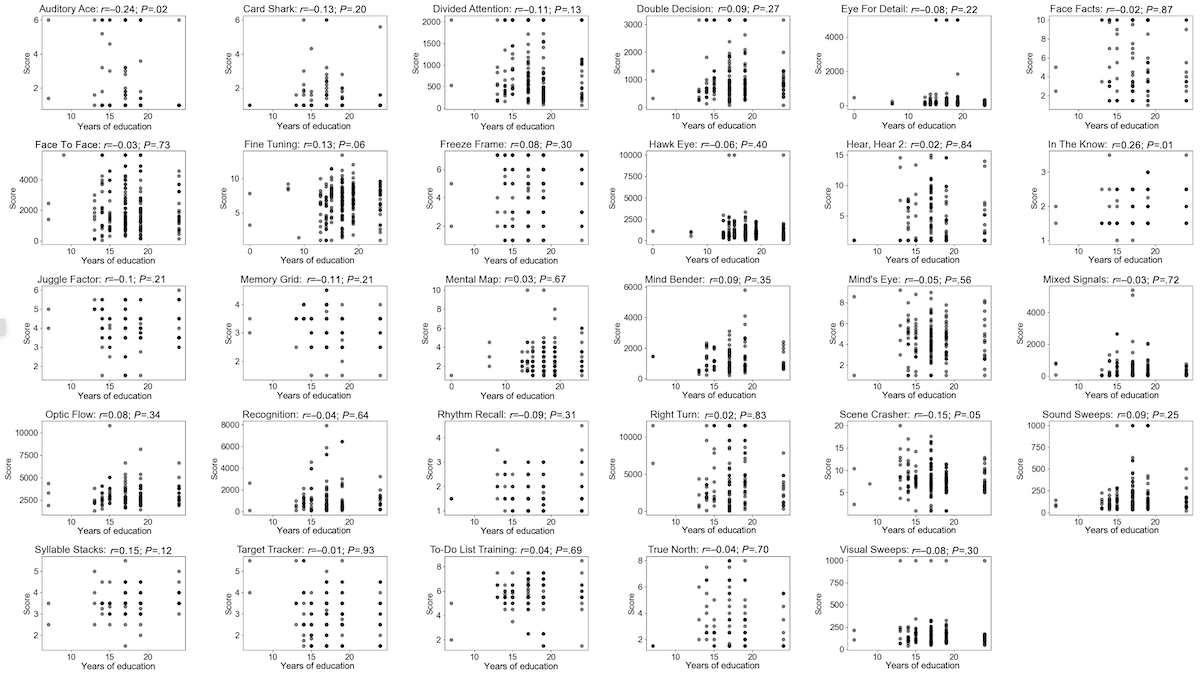
Association between performance and years of education for each assessment across registered participants.

The relationship between performance and years of education was not statistically significant for 93% (27/29) of the assessments (*P*>.05 in all cases), with the 2 exceptions being Auditory Ace (*P*=.02) and In The Know (*P*=.008; Figure 5).

**Figure 6:**
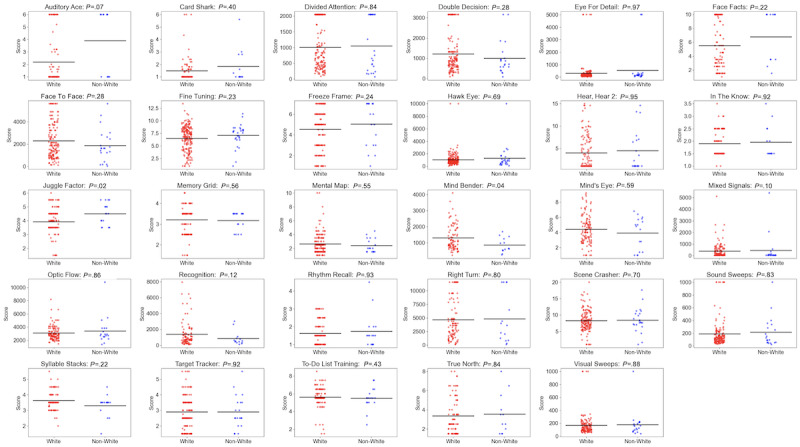
Association between performance and race (White vs people of color) for each assessment across registered participants.

The relationship between performance and race was not statistically significant for 93% (27/29) of the assessments (*P*>.05 in all cases), with the 2 exceptions being Juggle Factor (*P*=.02) and Mind Bender (*P*=.04; Figure 6).

### Composite z Score for “Completers”

The mean composite *z* score for participants who completed the 29 assessments was 0, with an SD of 0.19 (range –0.47 to 0.45; Table 3).

**Table 3:** Mean composite z scores for participants who completed the full battery of 29 assessments (N=104).

Composite *z* score	Completers, n (%)
–0.47 to –0.37	2 (1.9)
–0.37 to –0.27	3 (2.9)
–0.27 to –0.17	15 (14.4)
–0.17 to –0.07	23 (22.1)
–0.07 to 0.03	21 (20.2)
0.03 to 0.13	12 (11.5)
0.13 to 0.23	12 (11.5)
0.23 to 0.33	10 (9.6)
0.33 to 0.43	5 (4.8)
0.43 to 0.53	1 (1.0)

### Cognitive Profiles

**Figure 7:**
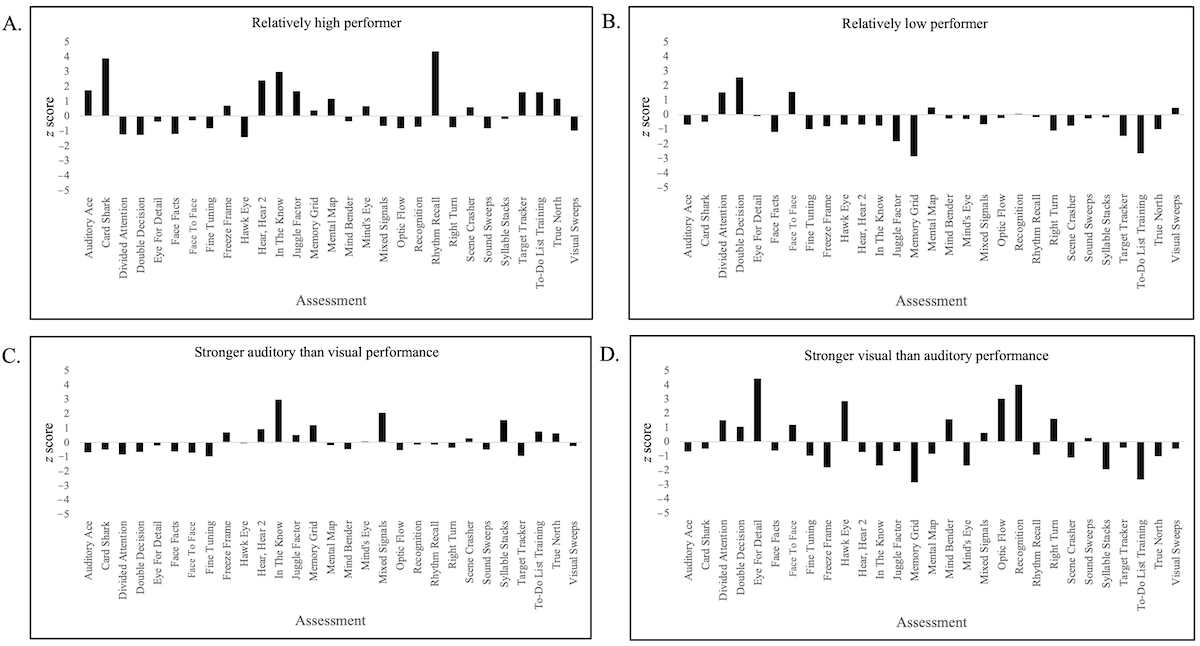
Examples of performance patterns across assessments revealing distinctive cognitive profiles, including (A) a relatively high-performing participant; (B) a relatively low-performing participant; (C) a participant with stronger auditory than visual performance; and (D) the reverse, showing a participant with stronger visual than auditory performance.

The pattern of performance across assessments revealed distinctive cognitive profiles (Figure 7), including relatively high-performing participants such as the user shown in Figure 7A (mean *z* score of 0.45 across all 29 assessments), relatively low-performing participants such as the user shown in Figure 7B (mean *z* score of –0.47), participants with stronger auditory than visual performance such as the user shown in Figure 7C (mean *z* score of 0.67 across the auditory assessments and mean *z* score of –0.27 across the visual assessments, with an overall composite of 0.09 across all 29 assessments), and participants with stronger visual than auditory performance such as the user shown in Figure 7D (mean *z* score of 0.72 across the visual assessments and mean *z* score of –1.22 across the auditory assessments, with an overall composite of 0.03 across all 29 assessments).

### Principal Component Analysis

The Bartlett sphericity test (*χ*^2^_28_=939.5; *P*<.001) and Kaiser-Meyer-Olkin (0.76) assumptions were not violated, suggesting that factor analysis was suitable for this data set. The 29 assessments loaded onto 4 factors (Table 4). Factor 1 reflected executive function or working memory demands, with small to large factor loadings for Scene Crasher, Juggle Factor, Card Shark, Target Tracker, Auditory Ace, True North, and Mental Map. Factor 2 reflected short-term memory demands, with large factor loadings for Syllable Stacks and Memory Grid. Factor 3 reflected delayed memory demands, with large factor loadings for Hear, Hear 2, and To-Do List Training. Factor 4 reflected processing speed demands, with small to large factor loadings for Divided Attention, Mixed Signals, Optic Flow, and Double Decision.

**Table 4:** Principal component analysis for participants who completed the full battery, indicating that the 29 assessments loaded onto 4 factors (N=104).

Assessment name	Factor 1 factor loadings	Factor 2 factor loadings	Factor 3 factor loadings	Factor 4 factor loadings
Scene Crasher	0.643301^a^	0.279054	0.225773	–0.014381
Juggle Factor	0.595876^a^	0.212351	0.36232	–0.248625
Card Shark	0.560295^a^	0.071172	0.040249	–0.301107
Target Tracker	0.509138^a^	0.203934	0.223545	–0.061977
Auditory Ace	0.503857^a^	0.122702	0.155117	–0.296731
True North	0.465121^a^	0.199453	0.353845	–0.025954
Mental Map	0.415206^a^	0.299156	0.249758	–0.030327
Face Facts	0.301677	0.341475	0.027211	–0.066479
Rhythm Recall	0.257117	0.006417	0.388292	–0.165549
Memory Grid	0.236313	0.530412^a^	0.226062	–0.070232
Hear, Hear 2	0.235846	0.047723	0.542699^a^	–0.163235
Fine Tuning	0.19339	0.389311	–0.13749	–0.08356
Mind’s Eye	0.157655	0.258577	0.389291	–0.197173
Syllable Stacks	0.083591	0.5406^a^	0.206336	0.039898
To-Do List Training	0.065038	0.346688	0.505778^a^	–0.147192
Mind Bender	–0.03505	–0.072113	–0.384683	0.042763
In The Know	–0.068834	0.020406	0.260689	–0.321159
Divided Attention	–0.099982	–0.107536	–0.13645	0.648114^a^
Right Turn	–0.117989	–0.1912	–0.312604	0.156221
Eye For Detail	–0.122413	–0.177566	–0.089855	0.190312
Optic Flow	–0.129046	–0.550209	–0.084751	0.492414^a^
Freeze Frame	–0.131735	0.240563	0.158117	–0.157661
Face To Face	–0.132008	–0.288762	–0.125521	0.091978
Visual Sweeps	–0.146627	–0.095615	–0.538311	0.075673
Sound Sweeps	–0.156355	0.038414	–0.473407	0.039435
Recognition	–0.225051	–0.581809	0.047476	0.380003
Mixed Signals	–0.274174	–0.071447	–0.108041	0.502077^a^
Double Decision	–0.302018	–0.345077	–0.286697	0.473593^a^
Hawk Eye	–0.310039	–0.337505	–0.143804	0.398793

^a^Factor loading of ≥0.4.

### Identifying Assessments With Potentially Useful Properties

From the total of 29 assessments, we defined a subset with potentially useful properties that may be combined into a core battery for future brain health evaluation. The assessments that were usable (either currently or with future modification of score bounds); informed by the principal component analysis; mostly sensitive to age; and insensitive to gender, race, and education were Scene Crasher, Syllable Stacks, To-Do List Training, Double Decision, Divided Attention, Eye For Detail, Face Facts, Face To Face, Fine Tuning, Mixed Signals, Optic Flow, Recognition, Sound Sweeps, and Target Tracker.

## Discussion

### Principal Findings

In this fully remote pilot study, we designed a digital toolbox of neurologically informed, adaptive visual and auditory brain health assessments and leveraged a robust, commercially available software infrastructure to deploy assessments on personal devices to participants en masse. A total of 29 modular assessments were successfully self-administered without oversight and showed reasonable usability, feasibility, and performance distributions. The pattern of performance across assessments revealed distinct cognitive profiles and loaded onto 4 factors reflecting executive function or working memory, short-term memory, delayed memory, and speed of processing.

A modular assessment framework offers researchers, clinicians, and administrators the flexibility to mix and match and concatenate any number of assessments into a custom battery of variable length, including as short as 3 minutes or as long as 2 hours. A total of 14 assessments in particular showed favorable psychometric properties that predictably scaled with age and were insensitive to differences in gender, level of education, and race. On the basis of these findings, a brief, “minimal” cognitive battery could include 2 assessments, such as Scene Crasher (for executive function) and Double Decision (for speed of processing), to quickly provide a “snapshot” of brain health for any user with an internet-connected device within approximately 6 minutes. A longer battery could include an assessment from each of the 4 factors (Scene Crasher, Syllable Stacks, To-Do List Training, and Double Decision) to offer a detailed look across indexes of speed, memory, and executive function. For a comprehensive evaluation, this set of 4 could be expanded with Divided Attention, Eye For Detail, Face Facts, Face To Face, Fine Tuning, Mixed Signals, Optic Flow, Recognition, Sound Sweeps, and Target Tracker to more finely understand a user’s cognitive strengths and weaknesses.

Most of the work to date has indicated that assessments are usable and feasible when taken in person under staff supervision. The findings of this study extend this work to show that assessments deployed in a real-world, unsupervised environment are similarly usable and feasible. A robust technical infrastructure allowed for the rapid collection of a normative data set within approximately 3 weeks and reached a wider audience across a range of personal devices at scale. This approach eliminated the need for in-person visits, travel, staff oversight, staff training, participant orientation, and manual scoring and interpretation. Participants could take the assessments in a comfortable, familiar location (potentially reducing test anxiety) on a known household device (reducing confusion), which are factors that may improve the integrity of assessment scores [95].

### Future Directions

Future studies should recruit a well-balanced and well-characterized cohort to cross-validate the assessments with established neuropsychological instruments and evaluate assessment validity (eg, convergent and discriminant), reliability (eg, test-retest), and performance differences across user devices (eg, web vs phone). We will also evaluate clinical utility by recruiting specific clinical populations to define cutoff points and clinically meaningful change.

### Potential Future Applications

Generally speaking, a battery of computerized, self-administrable assessments has the potential to provide a user-friendly and efficient way to assess the brain health and cognitive performance of diverse populations across various environments. In the following sections, we provide examples of real-world applications for remote computerized assessment that may be useful in the future.

#### Clinical Trials

Human clinical trials and longitudinal studies are the backbone of translational research. Reducing the costs of and improving access to assessments will be useful to a large number of investigators and help advance neurology, psychiatry, and neurocognitive care. Many neurological and psychiatric illnesses—particularly those in which the focus is on prevention of the onset of disease states (eg, dementia and schizophrenia)—will require trials with thousands of participants spanning multiple years. Such trials are very costly and may be cost prohibitive for low-cost interventions (eg, cognitive training, exercise, and nutrition or diet). A remotely delivered self-administered brain health assessment could reduce trial costs substantially, allowing trials to recruit more participants, test those participants more frequently, and follow participants for longer periods. In these studies, computerized assessments can be used to screen potential participants for eligibility; measure target engagement; aid in the interpretation of clinical trial results; evaluate the efficacy of promising interventions by comparing performance gains, plateaus, or declines; support trial continuity during pandemic-related lockdowns; and augment existing neuropsychological batteries that evaluate generalized patient benefit. The current development of the NIH Mobile Toolbox takes an initial step toward this future application of mobile assessments [96].

#### Optimization of Performance

Environments that require optimal performance abilities (eg, the military, professional sporting events, and high-risk working environments) are often cognitively demanding. Cognitive performance contributes to success in these environments, suggesting that measuring cognitive performance could contribute to selecting the right individuals for these environments or helping individuals in those environments optimize their cognitive performance. The military, sports leagues, and private companies often invest significant resources in measuring and training physical and skill performance for these duties, typically without corresponding strategies to measure and train brain performance. Remote assessments could identify the potential of each candidate by providing detailed profiles of strengths and weaknesses across cognitive domains, inform decisions regarding career placement for new and transitional personnel, track cognitive change over time due to learning and development, and assure that personnel are operating at optimal levels of performance to improve overall force effectiveness, readiness, resilience, and endurance throughout their career.

#### Diagnostics

A computerized assessment program has the potential to more closely align brain health evaluation with preventative protocols that other medical disciplines (such as cardiology) have already established to detect at-risk individuals. When cognitive evaluation is not a standard component of routine medical care, clinicians stand little chance of detecting early warning signs of medically serious cognitive impairments [97]. By the time a patient fails standard neuropsychological tests, extensive cortical and subcortical damage has occurred. That lag between the true onset (commonly initiated decades earlier) and measured “disease” onset largely stems from the lack of delivery of assessments across those decades that could have accurately indexed general brain health and tracked its (often) slow decline. In contrast, other fields such as cardiology have implemented simple and effective early detection measures to identify risk of cardiovascular disease for many decades via the measurement of indexes such as blood pressure, cholesterol, and weight and are estimated to have prevented millions of premature deaths [98]. Self-administered assessments may be used to provide an index of brain health or discriminate, stratify, or diagnose studied conditions [99]. A remote assessment battery is not intended to be a pure substitute for traditional neuropsychological testing but rather to allow physicians and health care plans to remotely screen patients and prioritize at-risk individuals for a full diagnostic workup, allowing for earlier detection of neurological and psychiatric issues. Providers will also be better equipped to track the developmental trajectories of these diseases throughout the preclinical, manifestation, and maturation stages. As brain health screening and tracking become a mainstay of routine medical practice, we can anticipate that the risks of brain-related conditions will be more reliably detected, treatments will be more often applied in prodromal individuals, and brain health management may minimize a continuance or recurrence of neurological issues.

### Limitations of This Study

A weakness of this study is that our convenience sample is not representative of the US population. Compared to US census data [100], the sample had a lower racial diversity (349/414, 84.3% White vs 76% White in the US census data) and was predominately female (265/414, 64% vs 50% in the US census data), older (5/414, 1.2% aged <18 years vs 22% in the US census data), and well educated (288/414, 69.6% with a bachelor’s degree or higher vs 34% in the US census data). Therefore, there are limitations in the generalizability of the findings. For this reason, the performance data should not be considered to support the full validation of these assessments, and certain preliminary conclusions from this data set (eg, insensitivity of the tests to educational level, gender, and race) may be revised with a representative population as part of a full validation study.

Other limitations include the dependence on digital literacy and access to technology and the low completion rate for the full battery of 29 assessments (likely due to the absence of monetary compensation for participation [101-103]). The testing environment was also difficult to standardize due to potential variability in the testing devices used, potential unanticipated midtest distractions and interruptions, and potential assistance from household members.

### Conclusions

Managing brain health in an accessible way is an unmet global challenge. The digital toolbox necessary to extend screening and monitoring into the home includes targeted assessments that are neurologically informed, usable, scalable, self-administrable, adaptive, and managed by a robust technical infrastructure. Furthermore, a modular assessment battery represents a substantial innovation over current batteries that are typically fixed in their organization and structure. This framework parallels clinical practice in which medical practitioners can select and order different combinations of blood panels “a la carte,” ranging from 1 or 2 tests to a more comprehensive investigative set depending on patient need.

A digital toolbox that adheres to the highest standards of assessment development will serve as a catalyst in translational neuroscience. By bridging the gap between advancements in basic science, community-based initiatives, and clinical expertise, this endeavor will expand the scope of screening and monitoring to include home-based settings. Once brain health management is understood and popularized, we anticipate that remote assessments will pave the way for improved care across the lifespan and health states.

## Appendix

Multimedia Appendix 1 Recruitment email, demographics questionnaire, display of assessment score, and engagement emails.

## Abbreviations

API: application programming interface
EXAMINER: Executive Abilities: Measures and Instruments for Neurobehavioral Evaluation and Research
NIH: National Institutes of Health
UFOV: Useful Field of View

## Data Availability

The data sets generated and analyzed during this study are available from the corresponding author upon reasonable request.
