# Supplementary material for: The Real-World Usability, Feasibility, and Performance Distributions of Deploying a Digital Toolbox of Computerized Assessments to Remotely Evaluate Brain Health: Development and Usability Study
Source: JMIR Form Res. 2024 May 13;8:e53623. doi: 10.2196/53623 (PMC11130778; doi:10.2196/53623)
Supplement: Multimedia Appendix 1 [file formative_v8i1e53623_app1.docx]

## Multimedia Appendix 1

**Figure S1.** Recruitment email sent to potential participants.


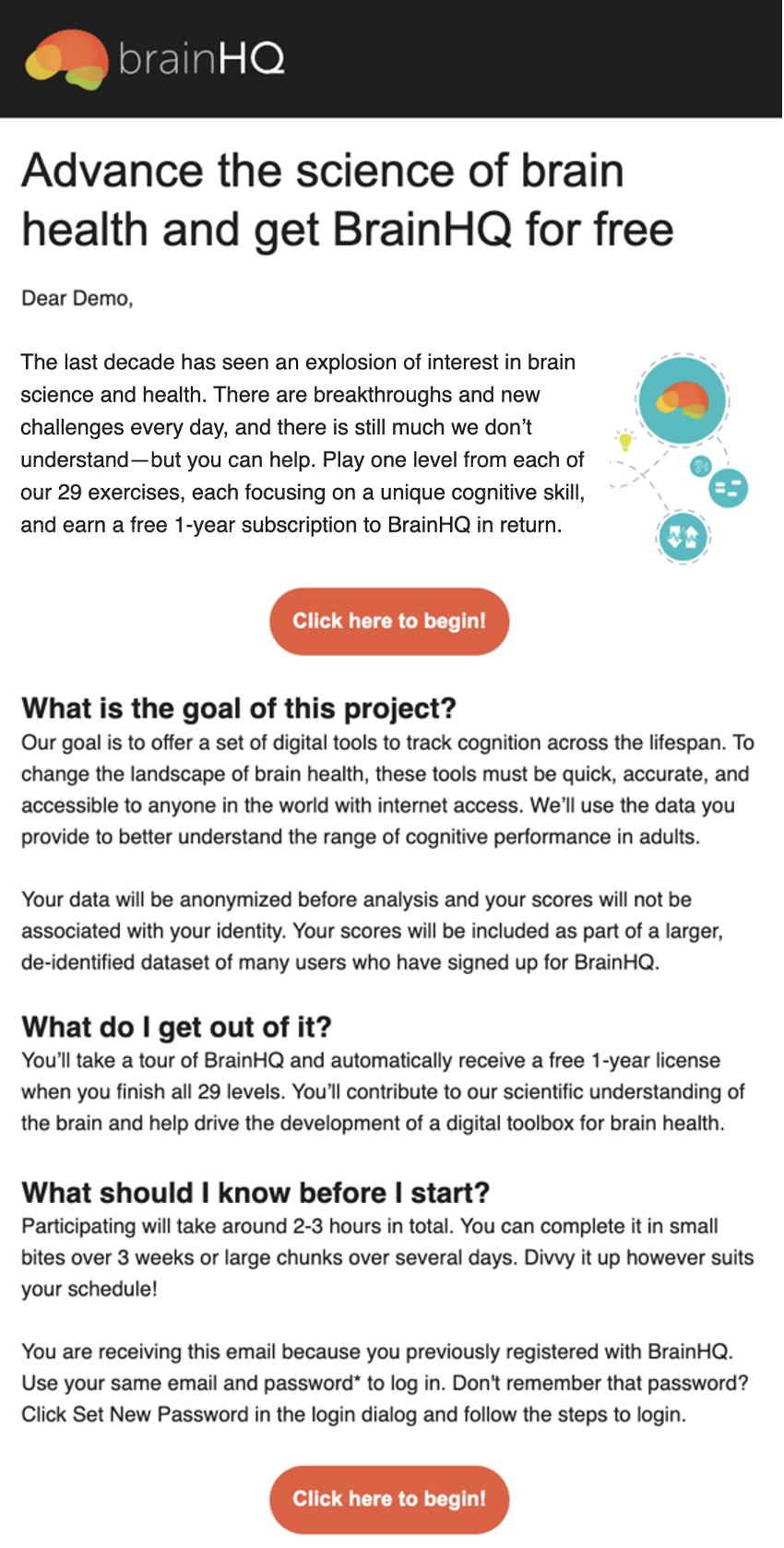


**Figure S2.** Upon login, participants who registered received a set of demographic questions for (A) age, (B) gender, (C) highest level of education attained, and (D) ethnicity.

**
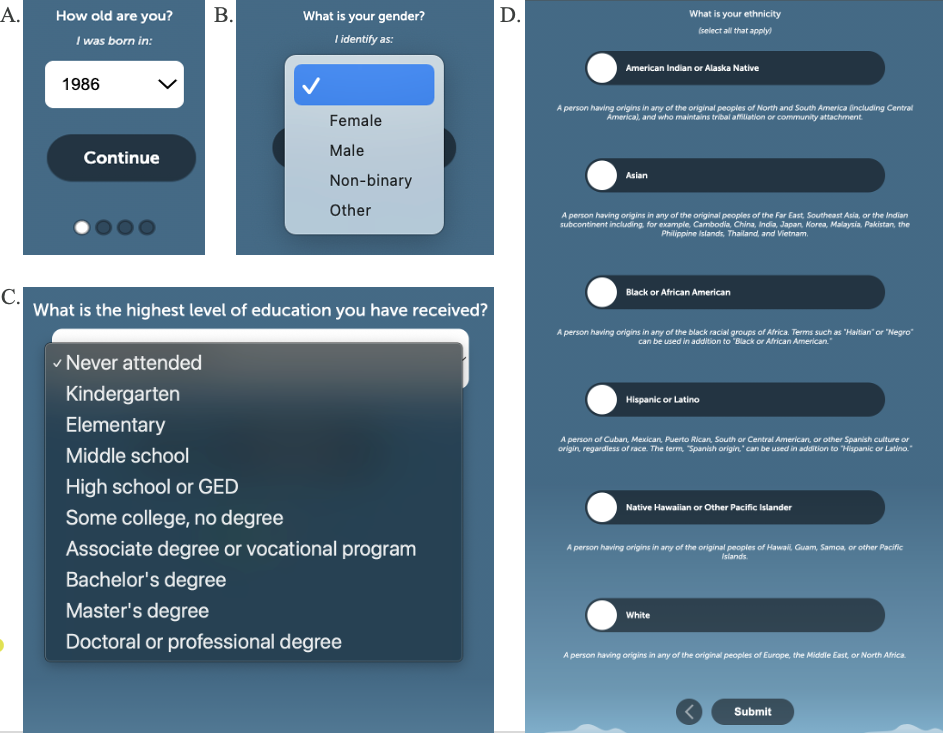
**

**Figure S3.** Participants were shown their score (a raw score and percentile) after completing each assessment.


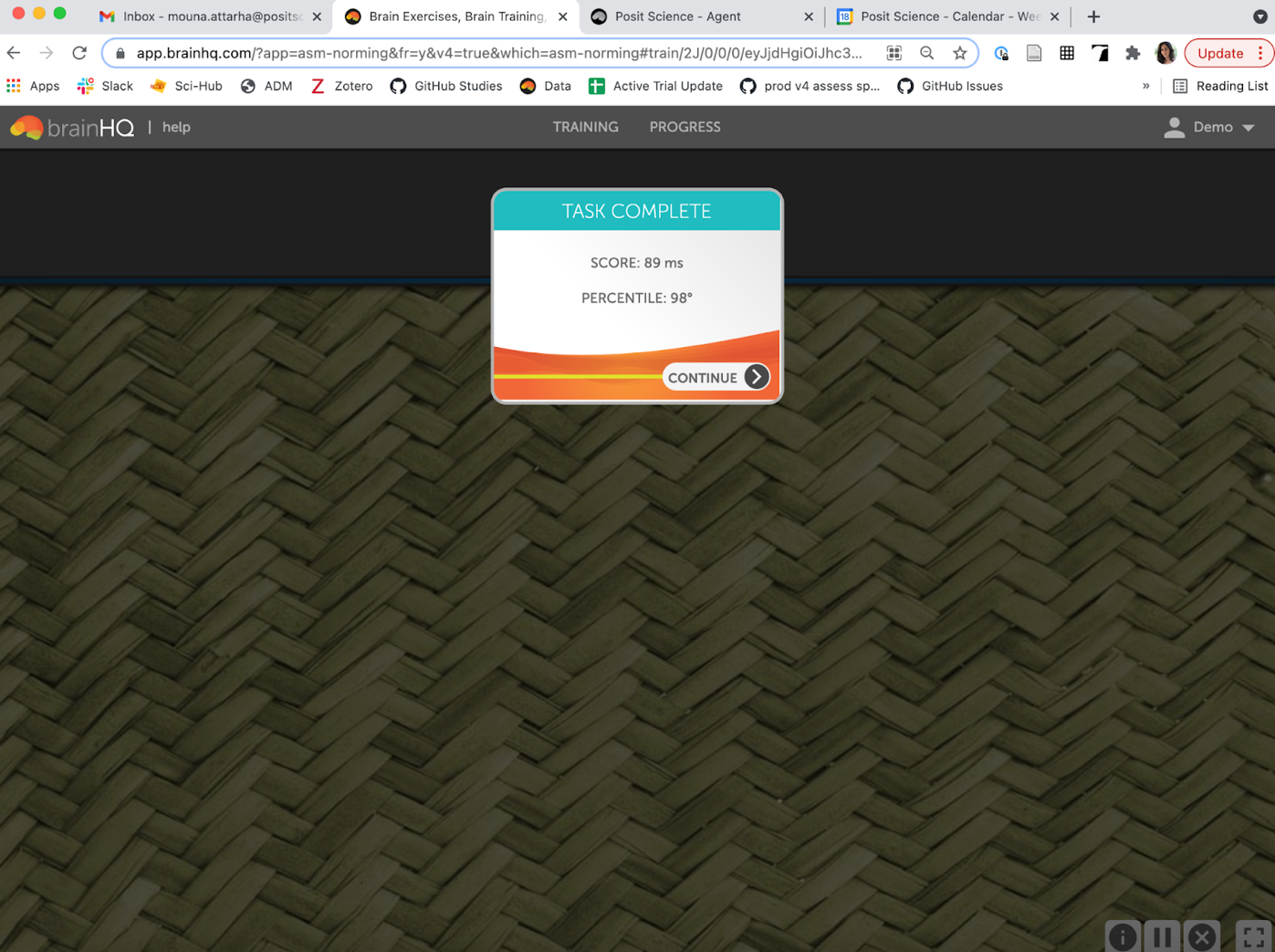


**Figure S4.** Engagement emails included (A) an introductory email sent upon participant registration on day 1, and reminder emails on (B) day 7, (C) day 14, and (D) day 20.

**
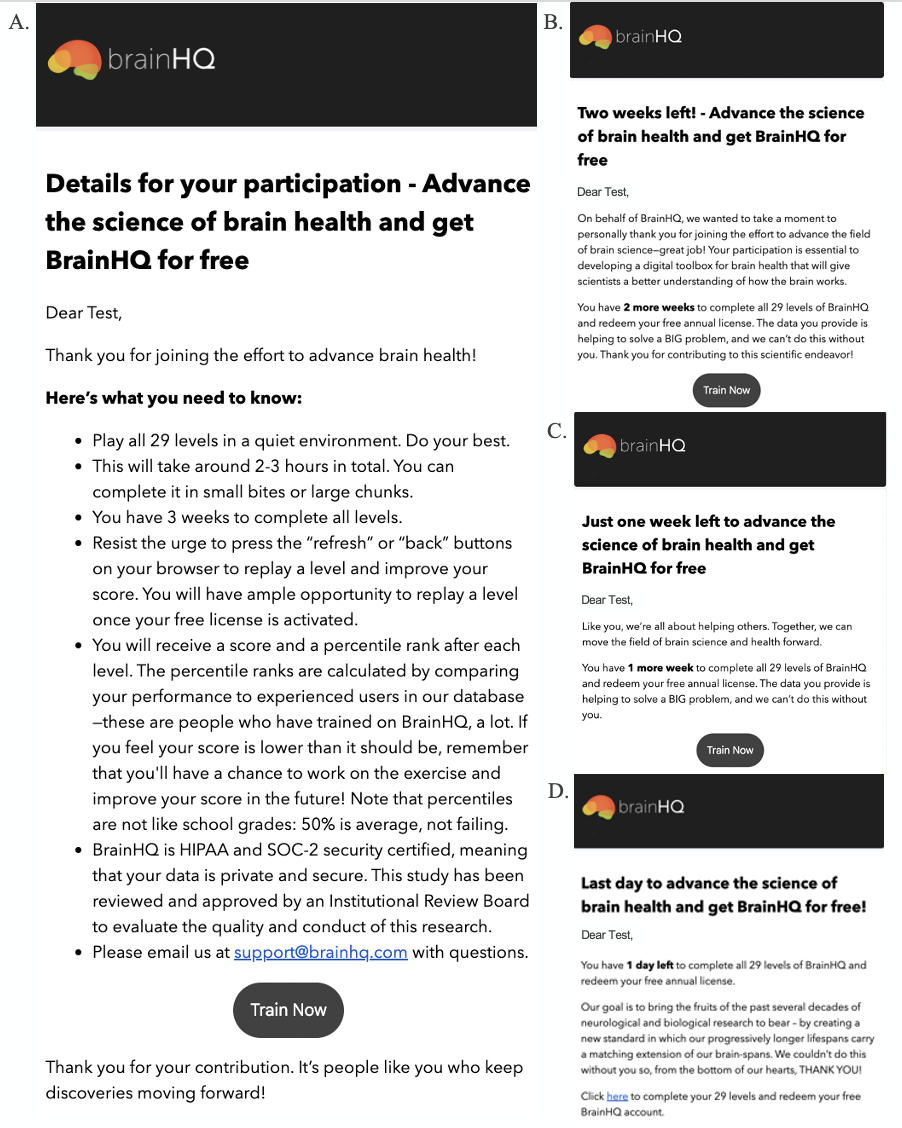
**
